# Physician Experience in Technical Success of Achieving NPVR ≥ 80% of High-Intensity Focused Ultrasound Ablation for Uterine Fibroids: A Multicenter Study

**DOI:** 10.3389/fmedt.2021.790956

**Published:** 2022-03-08

**Authors:** Xue Gong, Xinyue Zhang, Dang Liu, Chao Yang, Rong Zhang, Zhibo Xiao, Wenzhi Chen, Jinyun Chen

**Affiliations:** ^1^State Key Laboratory of Ultrasound in Medicine and Engineering, College of Biomedical Engineering, Chongqing Medical University, Chongqing, China; ^2^Department of Ultrasound Medicine, Mianyang Central Hospital, Mianyang, China; ^3^Department of Radiology, First Affiliated Hospital of Chongqing Medical University, Chongqing, China

**Keywords:** high-intensity focused ultrasound (HIFU), uterine fibroid, physician experience, training, technical success, non-perfused volume ratio (NPVR)

## Abstract

**Objective:**

To evaluate the experience of the physician of the technical success in high-intensity focused ultrasound (HIFU) ablation of uterine fibroids with a nonperfused volume ratio (NPVR) of at least 80%.

**Methods:**

Patients from a 20-center prospective study were enrolled in this study. In this study, among the 20 clinical centers, five centers had physician with >3 years of HIFU experience, and the other 15 centers initiated HIFU therapy <3 years, were defined as the experienced group and the inexperienced group, respectively. Technical success was defined as achieving NPVR ≥ 80% of uterine fibroids with no major complications and it was defined as the successful group; otherwise, it was defined as the unsuccessful group.

**Results:**

A total of 1,352 patients were included at the age of 41.32 ± 5.08 years. The mean NPVR (87.48 ± 14.91%) was obtained in the inexperienced group (86.50 ± 15.76%) and in the experienced group (89.21 ± 13.12%), respectively. The multivariate analysis showed that the volume of uterus, location of fibroids, and physician experience were significantly correlated with technical success (*p* < 0.05). In the experienced group, 82.20% of uterine fibroids obtained NPVR ≥ 80%, compared with 75.32% in the inexperienced group, and the difference was significant (*p* = 0.003). The technical success rate of the experienced group was 82.00% which was higher than 75.20% of the inexperienced group (*p* = 0.004).

**Conclusion:**

In technical success of achieving NPVR ≥ 80%, experience of the physician was positively correlated with technical success; NPVR and major complications for the inexperienced group were comparable to those of the experienced group from a clinical perspective; inexperienced physicians could reach NPVR ≥ 80% of sufficient ablation and were trustworthy in efficacy. Smaller uterus and fibroids of anterior wall were correlated with better technical success; experienced physicians still have better technical success when choosing patients with larger uterus, contributing to clinical decision-making and patient referral.

## Introduction

Uterine fibroids are benign lesions or neoplasms of the uterus that can enlarge the uterus and it is estimated that 25% of the patients can develop clinical symptoms (1, 2). There are many therapeutic strategies for uterine fibroids such as hysterectomy, myomectomy, laparoscopic myomectomy, and uterine artery embolization (UAE) therapy (3, 4). However, hysterectomy and myomectomy approaches are associated with a high rate of significant complications and weeks to recover (5, 6). There are also reports of prenatal uterine rupture after laparoscopic myomectomy (7) and major complication rates in UAE were estimated that range from 1 to 17% (8, 9). Every treatment approach should consider the age of the patient, childbearing plans, and the location and size of the leiomyoma (2, 10).

In the recent years, high-intensity focused ultrasound (HIFU) has been used in noninvasive ablation of uterine fibroids, which was a uterus-preserving therapy proved to be safe and effective (11, 12). However, HIFU technology was still in its infancy and it was not commonly used (13, 14). There were some differences in operation outcomes in surgeons according to experience of the physician and the surgical learning curve with increased surgeon experience (15, 16). The degree that impacts technical success in HIFU treatment was not known. In this study, we conducted a multicenter study to compare technical success in physician with different experience, in order to provide a basis for the clinical technical training, choices of physician, patient selection, and expanded application of HIFU technology.

## Materials and Methods

### Patients

From March 2011 to December 2013, the patients with uterine fibroids were enrolled in 20 clinical centers in China. Data of the patient were selected from the Multicenter Research Information System of Therapy of Uterine Fibroids (www.hifuctr.com) (software copyright certificate number: 2011SR094656). This multicenter study was approved by the Chinese Ethics Committee of Registering Clinical Trials (IRB approval number: ChiECRCT-2011034). Each patient signed a written informed consent before inclusion. The results of clinical efficacy have been reported (17).

Eligibility criteria for patients (17) were as follows: (1) Premenopausal women with completed planned families (and had no recent plan for a further pregnancy), (2) Imaging-confirmed diagnosis of uterine fibroids had any of the following indications for hysterectomy: (a) enlarged uterus (uterine volume ≥10 weeks of gestation); (b) menorrhagia and/or secondary anemia; (c) pelvic pain, urinary frequency, or constipation, (3) For patients with multiple fibroids, no more than three fibroids with minimal diameters of 2 cm based on abdominal ultrasound present, (4) Fibroids clearly imaged by abdominal ultrasound, and (5) For patients with abdominal surgical scars, the width of image blurring due to acoustic attenuation had to be <10 mm.

Exclusion criteria (17) were as follows: (1) Patients with uterine adenomyosis, (2) Previous myomectomy, (3) Concurrent pregnancy, (4) Pedunculated subserous or submucosal fibroids, (5) Any single fibroid >10 cm maximum in diameter, (6) Acute pelvic inflammation or uncontrolled systemic disease, and (7) Patients were unable to communicate with physicians or were unwilling to sign a written informed consent. Patients were provided with written information describing the potential risks and benefits including the potential impact on fertility and the risk of recurrence of symptoms.

### Eligibility Criteria for Physicians and Prior Training and Quality Control

A program of HIFU treatment training and qualification certification authorized by the Ministry of Health of China were required for physicians. All the operators were required to complete ablation treatment for 40–60 patients under guidance and supervision before entering the trial, with no major complications among treated patients, and a mean nonperfused volume ratio (NPVR) of treated uterine fibroids (NPVR ≥ 70%) (18).

### High-Intensity Focused Ultrasound Ablation Procedure

A single session of HIFU ablation was performed using the Model-JC/JC200 Focused Ultrasound Tumor Therapeutic System (Chongqing Haifu Medical Technology Corporation, Ltd, Chongqing, China). Equipment parameters used in this study were as follows: the frequency of the transducer was 0.8 MHz, the physical focal area was 1.5 × 1.5 × 10 mm, and the therapeutic power was 300–400 W. Standardized clinical program were used for physician training and clinical treatment. Patients placed in prone position on HIFU treatment bed and anterior abdominal wall was fully contact with degassed water. Fentanyl (0.8–1 μg/kg) and midazolam (0.02–0.03 mg/kg) were provided every 30–40 min for conscious sedation; all the patients kept awake or in light sleep with their breath and their breath, oxyhemoglobin saturation, heart rate, and blood pressure were monitored during the procedure. Monitored by real-time ultrasonography, the treatment used massive grayscale changes in the treated area as a measure of treatment effect. Adjust unit time dose according to tolerance of the patient and target grayscale changes. The sonication was terminated when the grayscale enhancement area covered the planned treatment area. Dose reference standard: total dose ≤ 150 kJ and treatment time ≤ 3 h. Post-operative patients were prone to observe 2 h.

### Magnetic Resonance Imaging Evaluation

All the patients underwent pelvic scans of MRI with 3.0 T MRI System before operation (GE Medical System, Milwaukee, Wisconsin, USA) including the T1-weighted imaging (T1WI), T2-weighted imaging (T2WI), and enhanced T1-weighted gradient-echo imaging contrast enhancement T1-weighted imaging (CE-T1WI) sequence. The three-dimensional diameters of dominant uterine fibroids and uterus were measured based on T2W images. Enhanced MR scans were performed again within 1 week after the treatment and used to measure the diameters of NPV. The fibroid volume and NPV were calculated by using the following equation: V = 0.5233 × D1 × D2 × D3 [longitudinal (D1), anteroposterior (D2), and transverse (D3)] (19). NPVR was the ratio of the volume of the nonperfusion area in the postablation to the volume of the fibroids. The signal intensity of the uterine fibroids was classified into three types according to the pretreatment T2WI: hypointense, isointense, and hyperintense (20).

### Technical Success

Technical success was defined as the NPVR of uterine fibroid at least 80% and with no major complications. Complications were recorded and graded using the guidelines of the Society of Interventional Radiology (SIR); classes A and B were considered to be minor complication and classes C to F were considered to be major complication (21, 22).

### Statistical Analysis

All the statistical analyses were performed by using the SPSS software version 22.0 (SPSS, IBM Corporation, Chicago, Illinois, USA). Continuous variables that followed the normal distribution were presented as the mean ± SD and the categorical variables were described by the total number. Continuous variables were compared by the independent *t*-test and categorical variables were compared by the chi-squared test. Binary logistic regression analysis was utilized for multivariate analysis. *p* < 0.05 was considered as statistically significant.

## Results

### Patients and Ultrasound Ablation Results

**Figure 1:**
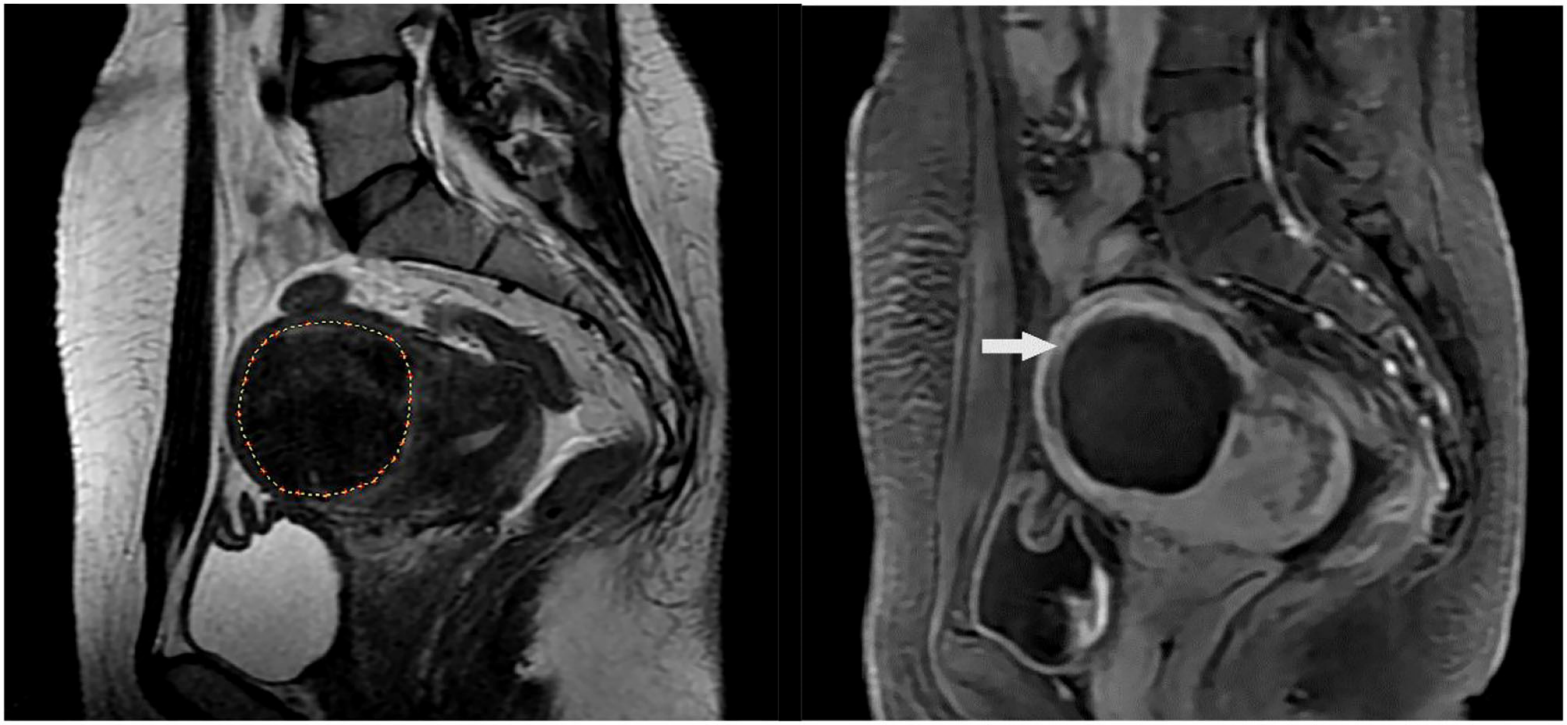
MR images of a 40-year-old woman with uterine fibroid. **(A)** T2-weighted image shows a single fibroid in the anterior wall of uterine before high-intensity focused ultrasound (HIFU) treatment; **(B)** Contrast-enhanced MRI shows nonperfused volume (NPV) in the uterine fibroid after HIFU treatment, NPV ratio (NPVR) was 94%. The white arrow indicates the nonperfusion volume (NPV) after treatment.

A total of 1,352 patients were included, with the exception of one patient who was diagnosed as diffuse intravenous leiomyomatosis during follow-up. The age of the patients was 41.32 ± 5.08 years (range: 23–57 years), body mass index (BMI) was 22.68 ± 2.99 kg/m^2^ (range: 16–43.3 kg/m^2^), the volume of dominant uterine fibroids was 104.86 ± 81.76 cm^3^ (range: 4.12–518.07 cm^3^), uterine volume was 292.32 ± 148.31 cm^3^ (range: 39.43–1,160.47 cm^3^), and the mean NPVR was 87.48% (range: 0.2–100%) (Table 1 and Figure 1).

**Table 1:** Baseline characteristics and the results of ultrasound ablation.

**Parameter**	**Data (*n* = 1,352)**
Age (year)	41.32 ± 5.08
BMI (kg/m^2^)	22.68 ± 2.99
Maximum diameter of fibroids (cm)	6.07 ± 1.57
Uterine volume (cm^3^)	292.32 ± 148.31
Volume of uterine fibroids (cm^3^)	104.86 ± 81.76
Ultrasonic power (W)	393.28 ± 22.23
Sonication time (sec)	1,174.71 ± 685.13
Total dose (kJ)	472.52 ± 276.29
NPV (cm^3^)	83.24 ± 72.59
NPVR (%)	87.48 ± 14.91

*NPVR, nonperfused volume ratio*.

### Comparison of Patients Between Different Experience Centers

Among the 20 clinical centers, five centers had five physicians with more than 3 years of HIFU experience as the experienced group and more than 50 cases in each of the five centers; the other 15 centers have 15 physicians <3 years of HIFU as the inexperienced group and only seven centers over 50 cases. In the inexperienced group, 12 hospitals have not carried out HIFU treatment before. A total of 863 patients with fibroids were in the inexperienced group and 489 patients with fibroids were in the experienced group accordingly. The independent sample *t*-test or the chi-squared test was used to analyze data and we found that no significant difference in age, BMI, position of uterus, number of fibroids, and location of fibroids (*p* > 0.05). The maximum diameter of fibroids, uterine volume, and uterine fibroid volume of the experienced group were larger than that of the inexperienced group (*p* < 0.05) and there were significant differences in type of fibroids and classification of T2WI signals intensity between two groups (*p* < 0.05). The percentage of submucosal fibroids in the experienced group was 14.93%, which was higher than that in the inexperienced group (6.84%) (*p* = 0.000). There were 430 fibroids with the hyperintense of T2WI, 35.38% in the experienced group, and 29.78% in the inexperienced group, respectively (*p* = 0.009) (Table 2).

**Table 2:** Comparison of patients with uterine fibroids between two groups of physicians.

**No. of patients**	**Inexperienced group** **(*n* = 863)**	**Experienced group** **(*n* = 489)**	***P***
Age (year)	41.35 ± 5.28	41.25 ± 4.71	0.701
BMI (kg/m^2^)	22.72 ± 3.10	22.61 ± 2.80	0.520
Maximum diameter of fibroids (cm)	5.95 ± 1.55	6.28 ± 1.60	0.000
Volume of uterine fibroids (cm^3^)	99.25 ± 78.19	114.76 ± 86.92	0.001
Volume of uterus (cm^3^)	278.04 ± 142.02	317.38 ± 155.90	0.000
Position of uterus			0.160
Anteverted	608 (70.45)	362 (74.03)	
Retroverted	255 (29.55)	127 (25.97)	
Number of fibroids			0.151
Solitary	656 (75.90)	354 (72.39)	
Multiple	208 (24.10)	135 (27.61)	
Location of fibroids			0.413
Anterior wall	396 (45.89)	210 (42.94)	
Posterior wall	229 (26.54)	148 (30.27)	
Lateral wall	185 (21.44)	97 (19.84)	
Fundus	53 (6.14)	34 (6.95)	
Type of fibroids			0.000
Intramural	667 (77.29)	330 (67.48)	
Submucosal	59 (6.84)	73 (14.93)	
Subserosal	137 (15.87)	86 (17.59)	
T2WI sign intensity			0.009
Hypointense	463 (53.65)	220 (44.99)	
Isointense	143 (16.57)	96 (19.63)	
Hyperintense	257 (29.78)	173 (35.38)	

### Comparison of Factors Between the Unsuccessful Group and the Successful Group

A total of 1,050 patients with NPVR ≥ 80% of uterine fibroids with no major complications were in the successful group, while 302 patients were in the unsuccessful group. Analysis of two groups of characteristics from experience of the patients and physicians, the independent *t*-test and the chi-squared test were used in the univariate analysis. The volume of uterus, location of fibroids, and physician experience were statistically significant (*p* < 0.05) (Table 3).

**Table 3:** The univariate analysis to evaluate the factors between the unsuccessful group and the successful group.

**Factors**	**Unsuccessful group** **(*n* = 302)**	**Successful group** **(*n* = 1,050)**	***P***
Age (year)	41.38 ± 0.31	41.51 ± 0.15	0.884
BMI (kg/m^2^)	22.66 ± 0.18	22.50 ± 0.09	0.523
Maximum diameter of fibroids (cm)	5.90 ± 0.09	6.06 ± 0.05	0.084
Volume of uterine fibroids (cm^3^)	91.07 ± 4.21	98.32 ± 2.59	0.068
Volume of uterus (cm^3^)	297.56 ± 9.00	275.27 ± 4.50	0.036
Position of uterus			0.498
Anteverted	212 (70.2)	758 (72.2)	
Retroverted	90 (29.8)	292 (27.8)	
Number of fibroids			0.721
Solitary	223 (73.8)	786 (74.9)	
Multiple	79 (26.2)	264 (25.1)	
Location of fibroids			0.001
Anterior wall	105 (34.8)	501 (47.7)	
Posterior wall	101 (33.4)	276 (26.3)	
Lateral wall	72 (23.8)	210 (20.0)	
Fundus	24 (7.9)	63 (6.0)	
Type of fibroids			0.264
Intramural	221 (73.2)	776 (73.9)	
Submucosal	24 (7.9)	108 (10.3)	
Subserosal	57 (18.9)	166 (15.8)	
T2WI sign intensity			0.148
Hypointense	110 (36.4)	320 (30.5)	
Isointense	50 (16.6)	189 (18.0)	
Hyperintense	142 (47.0)	541 (51.5)	
Physician experience			0.003
Inexperienced group	215 (71.2)	648 (61.8)	
Experienced group	87 (28.8)	402 (38.2)	

### Logistic Regression Analysis of Technical Success of Achieving NPVR ≥ 80%

In Table 4, the multivariate logistic regression analysis showed that the volume of uterus, location of fibroids, and physician experience were significantly correlated with technical success. The volume of uterus was negatively correlated with technical success and fibroids of anterior wall and experienced physicians were positively correlated with technical success (*p* < 0.05).

**Table 4:** The multivariate binary logistic regression analysis to evaluate the correlation of technical success with the significant factors of the univariate analysis.

**Variables**	**B**	**S.E**.	**Wald**	**P**	**ORs [Exp (B)]**	**95% CI**	
						**Lower**	**Upper**
Volume of uterus	−0.001	0.000	6.245	0.012	0.999	0.998	1.000
Location of fibroids			16.390	0.001			
Posterior wall	−0.587	0.159	13.535	0.000	0.556	0.407	0.760
Lateral wall	−0.472	0.175	7.288	0.007	0.624	0.442	0.879
Fundus	−0.597	0.265	5.067	0.024	0.550	0.327	0.926
Inexperienced group	0.493	0.145	11.573	0.001	1.637	1.232	2.174

### Comparison of Technical Success Between two Groups

There was no statistically significant difference in major adverse events between the inexperienced group and the experienced group (*p* = 1.000). NPVR was 87.48 ± 14.91% in the inexperienced group (86.50 ± 15.76%) and in the experienced group (89.21 ± 13.12%), respectively. In the experienced group, 82.20% of uterine fibroids obtained NPVR ≥ 80%, compared with 75.32% in the inexperienced group, and the difference was significant (*p* = 0.003). The rate of the technical success accounts for 82.00% in the experienced group higher than the rate of the technical success accounts for 75.20% in the inexperienced group (*p* = 0.004) (Table 5).

**Table 5:** Comparison of the success indicators between two groups.

**Success indicators**	**Inexperienced group** **(*n* = 863)**	**Experienced group** **(*n* = 489)**	***P***
NPVR (%)	86.50 ± 15.76	89.21 ± 13.12	0.001
No. of NPVR ≥ 70%	758 (87.83)	449 (91.82)	0.023
No. of NPVR ≥ 80%	650 (75.32)	402 (82.20)	0.003
Major adverse events	2 (0.23)	1 (0.20)	1.000
Technical success	649 (75.20)	401 (82.00)	0.004

*NPVR, nonperfused volume ratio*.

### Adverse Events

Following the SIR classification as shown in Table 6, a total of 1,068 (78.99%) adverse events were classified as class A and all of these adverse events recovered spontaneously in few days without any treatment. The unsuccessful group and the successful group showed significant difference with vaginal bleeding and discharge (*p* = 0.028); lower abdominal pain, lumbar and back (sacrum) pain, leg numbness/pain, weakness in lower limb, blurred vision, dizziness and headache, pain, and distension of anus showed no significant differences (*p* > 0.05). Adverse events classified as class B were 469 (34.69%); lower abdominal pain was significant difference (*p* = 0.000); sacrum/buttock pain, vaginal bleeding and discharge, leg numbness/pain, weakness in lower limb, fever, nausea and vomiting, hematuria, respiratory tract infection, and urinary retention were no statistical significance in the unsuccessful group and the successful group (*p* > 0.05). Major adverse events attributable to the intervention occurred in three (0.22%) patients during 30 days follow-up period and were classified as class C; these three patients were in the unsuccessful group and were second-degree skin burns that increased the level of care and did not lead to substantial morbidity and disability; skin burns were significant indigenous statistical difference between the unsuccessful group and the successful group (*p* = 0.001).

**Table 6:** Summary of postprocedural adverse effects.

**SIR classification**	**Adverse event**	**Unsuccessful group** **(*n* = 302)**	**Successful group** **(*n* = 1,050)**	**P**
Class A	Lower abdominal pain	154 (0.51)	502 (0.48)	0.329
	Lumbar and back (sacrum) pain	24 (0.08)	96 (0.09)	0.520
	Vaginal bleeding and discharge	43 (0.14)	208 (0.20)	0.028
	Leg numbness /pain	2 (0.01)	8 (0.01)	0.859
	Weakness in lower limb	1 (0.00)	7 (0.01)	0.503
	Dlurred vision	0 (0.00)	11 (0.01)	0.074
	Dizziness and headache	1 (0.00)	0 (0.00)	0.062
	Pain and distension of anus	2 (0.01)	9 (0.01)	0.740
Class B	Lower abdominal pain	23 (0.08)	167 (0.16)	0.000
	Sacrum/buttock pain	4 (0.01)	28 (0.03)	0.176
	Vaginal bleeding and discharge	42 (0.14)	153 (0.15)	0.772
	Leg numbness /pain	3 (0.01)	21 (0.02)	0.243
	Weakness in lower limb	0 (0.00)	1 (0.00)	0.592
	Fever	0 (0.00)	2 (0.00)	0.448
	Nausea and vomiting	4 (0.01)	17 (0.02)	0.715
	Haematuria	1 (0.00)	0 (0.00)	0.062
	Respiratory tract infection	0 (0.00)	1 (0.00)	0.592
	Urinary retention	0 (0.00)	2 (0.00)	0.448
Class C	Skin burns	3 (0.01)	0 (0.00)	0.001
Class D	–	–	–	–
Class E	–	–	–	–

## Discussion

High-intensity focused ultrasound therapy is an uterine-sparing alternative that reported to be safe, effective, and highly acceptable to patients in the treatment of fibroids (11). HIFU treatment has a potential impact on improving the quality of life on patients (12). The United States Food and Drug Administration (FDA) approved the focused ultrasound therapy as entirely a noninvasive approach in treatment of symptomatic uterine fibroids in 2004. HIFU has now become the preferred therapy of fibroids in some centers in China (17, 23). As a new technology for the treatment of uterine fibroids, physician training is the key way to popularize clinical applications and benefit more patients. Understanding the impact of experience of the physicians on technical success is an essential tool to improve safety of the patient and operation training.

Nonperfused volume ratio was directly related to the long-term relief of clinical symptoms, was as large as possible, and was positively associated with symptom control that was ultimately considered as a technical parameter for treatment success in focused ultrasound (17, 24, 25). Early in the application of HIFU technology, NPVR up to 25% can achieve the purpose of clinical symptoms relief. However, it was reported that the reintervention rate was 66.7% when median NPV of 36.4% of all the fibroid tissue volume (26, 27). The regression analysis indicated that when the NPVR directly posttreatment achieved 60%, the probability of undergoing an additional treatment was 10% at a mean 2 years after HIFU treatment of uterine fibroids; the results were comparable to those of myomectomy (14, 28). Two other studies indicated that when NPVR was higher than 70%, the 2-year clinical cumulative recurrence rates after HIFU were lower when compared to myomectomy (29, 30). In earlier studies, the prediction of immediate NPV ratio of more than 60%, which was used as an outcome evaluation index for HIFU treatment of uterine fibroids; on this basis, the technical requirements for operation training were NPVR reached at 70%. Park et al. (31) reported that HIFU ablation of uterine fibroids achievement of an immediate NPVR of at least 80% was safe, with greater tumor volume shrinkage compared with cases with a lower NPVR. Recently, some scholars have set NPVR at least 80% as a measure of sufficient ablation indexes in HIFU treatment of uterine fibroids (32). In addition, some scholars investigated MRI screening parameters for predicting an NPV ratio of at least 90% (33, 34). Among the 20 centers in this study, we aimed to obtain a higher NPVR on the premise of safety, so as to improve the clinical effect of HIFU ablation of uterine fibroids. The standard for HIFU ablation of uterine fibroids was to achieve an average of NPVR ≥ 70% after training and all the centers reached this technical requirement. With the improvement of clinical protocols and more technical experience of physician, the higher NPVR may result in better patient outcomes. Therefore, NPVR ≥ 80% was selected as the technical success criteria of HIFU ablation for uterine fibroids in this study. The results of both the groups had achieved the average NPVR ≥ 80% and NPVR was higher in the experienced center group.

In this study, the major adverse events in the inexperienced group were 0.23% and the major adverse events in the experienced group were 0.20%. There was no statistical significance between the two groups. Adverse events of class A need no therapy and no consequences and adverse events of class B need nominal therapy and no consequence, but need only overnight admission for observation; adverse events of class C require therapy and minor hospitalization (<48 h) (21). The proportion of vaginal bleeding and discharge and lower abdominal pain in the successful group was higher than that in the unsuccessful group in grade class A and class B adverse events, of the three patients in the unsuccessful group, NPVR ≥ 80% in two patients and NPVR <80 % in one patient, indicating that more observation and nursing were needed when the NPVR ≥ 80%. In clinical practice, vaginal bleeding and discharge were related to the location of uterine fibroids and vaginal bleeding and discharge prone to occur in submucosal fibroids after thermal injury; when the NPVR was higher, the boundary was close to the normal tissue, and it was prone to normal tissue thermal damage and stimulation pain of submucosal and subserosal fibroids; this also explained that class B had more lower abdominal pain in the successful group than in the failed group (35, 36). The experienced group was better than the inexperienced group in NPVR, which indicated that the physicians with more experience had better understanding on controlling the boundary in HIFU ablation of uterine fibroids. All the inexperienced group and the experienced group reached NPVR ≥ 80%; the difference between the two groups was only 2.7%, means that the inexperienced group was comparable to those of the experienced group from a clinical perspective in NPVR. On one hand, it shows that HIFU technology is easy to learn and promote and inexperienced physicians can achieve sufficient ablation level. On the other hand, experienced physicians in clinical practice have provided sufficient dose, sufficient ablation level, and remission of clinical symptoms when NPVR reaches 80% and experienced physicians will not blindly improve NPVR considering the safety boundary.

In terms of HIFU ablation technology, previous studies in comparison to the lesions located at the superficial region, lesions located deeper required more energy to achieve the same volume of necrotic tissue (37, 38). HIFU ablation of uterine fibroids was affected by many factors. The signal hypointense on T2WI of uterine fibroids, large size of uterine fibroids, or anteverted uterine could be more easily ablated with high ablation efficiency (37, 39). The technical indication for HIFU ablation of uterine fibroids was that lesions can be localized by ultrasound equipped in HIFU system. However, the selection of clinical cases was still influenced by the experience of the physician and his confidence in the effectiveness of the new technology. In this study, maximum diameter of fibroids, volume of uterine fibroids, and uterine volume in the experienced group were larger; the proportion of submucosal fibroids and fibroids with T2WI hyperintense signal were also higher than that of the inexperienced group; volume of uterus was negatively correlated with technical success. The reason may be that inexperienced physicians lack technical judgment ability. On the other hand, inexperienced physicians have less confidence in HIFU technique than traditional surgery and they were more inclined to recommend traditional surgery for patients with large uterine volume. As a result, inexperienced physicians in the selection of patients were inevitably biased. It was believed that with the accumulation of experience of the physicians, more patients can be selected to receive HIFU treatment. Another notable truth was that with HIFU, most clinical reports of HIFU treatment for fibroids were from China, while many other countries were still considered limit in clinical experience; studying the influence of experience of the physicians on the outcome of HIFU treatment of uterine fibroids could increase the trust in HIFU in centers that lack of HIFU experience (13). The technical success rate of the experienced group was superior to the inexperienced group in technical success rate that also verified the hypothesis of “practice makes perfect” (40). Therefore, with the accumulation of experience, higher NPVR can be obtained on the premise of safety, so as to improve the clinical effect of HIFU ablation of uterine fibroids.

The nature of the hospitals cannot be ignored in analyzing the factors that affect the success of HIFU technology; the influence of the level and nature of the hospitals on technical success can be studied (40, 41). The level and nature of the hospitals reflect the comprehensive level and feature of the hospitals. There may also be differences in the source of patients and the expertise of physicians. Therefore, the influence of HIFU on the technical success needs further study. No study showed the effect of experience of the physicians on the long-term prognosis and reintervention of patients (14, 41). Thus, prospective studies with larger sample sizes were needed to investigate the long-term results including long-term symptom relief, the recurrence rate, and the reintervention rate.

There were some limitations in this study. A total of 20 clinical centers in China were selected, but it was not clear whether consistent results can be obtained in international multicenters. There were many differences in medical education model, medical system, and understanding of diseases of the patient, so whether training programs were applicable and whether standards for technical success were appropriate that still need to be further studied. Adverse events during long-term follow-up were not evaluated, large-scale investigation with long-term follow-up was crucial, and there was a risk of uterine rupture after HIFU treatment (42, 43).

This study has several important implications. The relationship between experience of the physician and patient outcomes was an ancient topic. However, this study proved that HIFU was safe and all the centers in this study could reach the basic requirements of NPVR ≥ 70%; when NPVR set at ≥ 80%, the treatment result was better than that of myomectomy (14, 28, 44, 45). Notably, the inexperienced physicians could reach NPVR ≥ 80% and the experienced group was better than the inexperienced group in NPVR and technical success, indicating that HIFU technology was stable and easy to learn and can tend to be better technical success with better experience, which was worth considering doing HIFU physicians engaged in long-term development. Furthermore, appropriate patient selection was a significant factor in reducing the risk of an unsuccessful outcome of HIFU treatment of uterine fibroids (39, 46); this study discussed characteristic factors of the patient influencing technical success of HIFU ablation for uterine fibroids and whether there were differences in patient selection between the inexperienced physicians and the experienced physicians that provide a certain basis for the choice of patients.

In conclusion, when the treatment standard of HIFU for uterine fibroids is NPVR ≥ 80 %, the two groups of physicians can complete this standard; the inexperienced physicians are also trustworthy in the sufficient ablation for HIFU ablation of uterine fibroids. NPVR ≥ 80% is a standard that is worth being used in HIFU treatment of uterine fibroids. HIFU treatment of uterine fibroids is affected by multiple factors, experienced physicians, smaller volume of uterus, and fibroids of anterior wall that are easier to ablate with higher treatment success. In this study, experienced physicians still achieved better technical success in selecting patients with larger uterus for treatment, providing a basis for physicians with different experience to select patients. In addition, in the selection of indications, with the rich of experience of the physician, more patients with uterine fibroids are suitable for HIFU ablation treatment.

## Data Availability Statement

The raw data supporting the conclusions of this article will be made available by the authors, without undue reservation.

## Ethics Statement

The studies involving human participants were reviewed and approved by Chinese Ethics Committee of Registering Clinical Trials (IRB approval number: ChiECRCT-2011034). The patients/participants provided their written informed consent to participate in this study. Written informed consent was obtained from the individual(s) for the publication of any potentially identifiable images or data included in this article.

## Author Contributions

XG, XZ, DL, CY, RZ, ZX, WC, and JC provide methods and ideas. XG, XZ, and JC visited and analyzed the data. XG and JC wrote and revised the manuscript. All authors contributed to the article and approved the submitted version.

## Funding

This study was supported by the Chongqing Medical University Intelligent Medicine Project (Grant Number ZHYX2019007).

## Conflict of Interest

The authors declare that the research was conducted in the absence of any commercial or financial relationships that could be construed as a potential conflict of interest.

## Publisher's Note

All claims expressed in this article are solely those of the authors and do not necessarily represent those of their affiliated organizations, or those of the publisher, the editors and the reviewers. Any product that may be evaluated in this article, or claim that may be made by its manufacturer, is not guaranteed or endorsed by the publisher.
